# Enhancing low-dose CT denoising via multi-view knowledge transfer without paired data

**DOI:** 10.3389/fradi.2026.1691258

**Published:** 2026-05-08

**Authors:** Yueyang You, Li Xu, Gen Wei, Fuzhou Hua, Yingbo Hu

**Affiliations:** 1 The Second Affiliated Hospital, Jiangxi Medical College, Nanchang University, Nanchang, China; 2 Teaching Office, Affiliated Hospital of Jiangxi University of Chinese Medicine, Nanchang, China

**Keywords:** CT denoising, knowledge transfer, low-dose CT, multi-view learning, unpaired data

## Abstract

**Introduction:**

Despite remarkable advancements in deep learning for low-dose computed tomography (LDCT) denoising, two significant challenges persist: (i) the requirement for paired LDCT and high-dose computed tomography (HDCT) images, which are often impractical to obtain in clinical settings; and (ii) the tendency of existing methods to train models using a single axial view, thereby overlooking complementary information from other views and consequently limiting their performance.

**Methods:**

To address these issues, we propose a Multi-view-to-Single Knowledge Transfer (MvSKT) framework for unpaired LDCT denoising. Our approach involves splitting the 3D unpaired computed tomography (CT) data into 2D images from various views, including axial, sagittal, and coronal. This allows us to train three view-independent 2D GAN models in an unsupervised manner. By stacking successive 2D outputs from each view-independent model into a volumetric format and splitting them into axial-view images, we generate multiple complementary predictions for each axial CT image. Leveraging these predictions as priors, we transfer multi-view knowledge to a single-view model through pseudo-supervision. This process involves fusing multiple view-complementary predictions into reliable pseudo-images using a cycle-consistency-weighted method.

**Results:**

Extensive experiments on the AAPM-Mayo dataset demonstrate that MvSKT outperforms other unpaired denoising methods and even achieves performance comparable to supervised approaches.

**Discussion:**

Consequently, the MvSKT framework effectively harnesses multi-view information from unpaired data to enhance LDCT denoising without the strict requirement of paired clinical data.

## 1 Introduction

Computed tomography (CT) is as a cornerstone of clinical diagnostics, providing vital imaging for medical assessment. However, its widespread use has raised concerns about exposure to associated X-ray radiation, prompting a shift towards low-dose CT (LDCT) to mitigate health risks (1). Despite its benefits, LDCT is prone to increased quantum noise and streak artefacts, which significantly impair image quality. Addressing this, the development of effective noise reduction techniques for LDCT has emerged as a critical research focus, aiming to enhance image quality while minimizing radiation exposure.

Over the past decades, numerous LDCT denoising approaches have been proposed, with recent advancements largely attributed to deep neural networks (DNNs) due to their robust ability to learn from raw data (2–23). Most of these methods are designed to learn a non-linear mapping from LDCT to high-dose CT(HDCT) images in a supervised manner (5, 14, 18, 23). However, acquiring real paired LDCT–HDCT data remains a significant challenge, as it necessitates sequential scanning of the same patient at both dose levels, leading to excessive radiation exposure. Consequently, existing supervised learning approaches often rely on simulated LDCT data derived from HDCT data or on data obtained from animals (24) and cadavers (25). Although these methods have shown notable performance, they are constrained by the limitations of simulated or impractical paired CT data, which has become a bottleneck in advancing LDCT denoising.

In contrast, unpaired LDCT and HDCT data are more readily available. Developing denoising methods that do not rely on paired data can leverage the rich information contained within large volumes of unpaired data, offering a more practical approach to LDCT denoising. Existing unpaired LDCT denoising methods typically focus on single axial-view processing, thereby neglecting the valuable complementary information from other 3D CT views, such as coronal and sagittal (9, 13, 26–28). 3D medical volumetric data, including CT and MRI, can be transformed into a series of 2D images across multiple views, providing diverse information at the view level. Due to multi-view consistency, LDCT denoising can be performed from different views for each voxel in the 3D LDCT volume. Moreover, noise patterns vary across different views, highlighting noise disparity in various views. Based on these observations, we posit that leveraging multi-view information can significantly enhance LDCT denoising performance.

A straightforward approach to using multi-view information for unpaired 2D LDCT denoising involves a multi-model ensemble, where three separate unsupervised models are trained on images from axial, coronal, and sagittal views, and their 2D predictions are fused into 3D volume. However, this method is inefficient for clinical examination, as it requires fusing multi-view 2D predictions into 3D results and then splitting them back into 2D for radiological review. Moreover, employing multiple models increases inference time and adds to deployment complexity.

**Figure 1:**
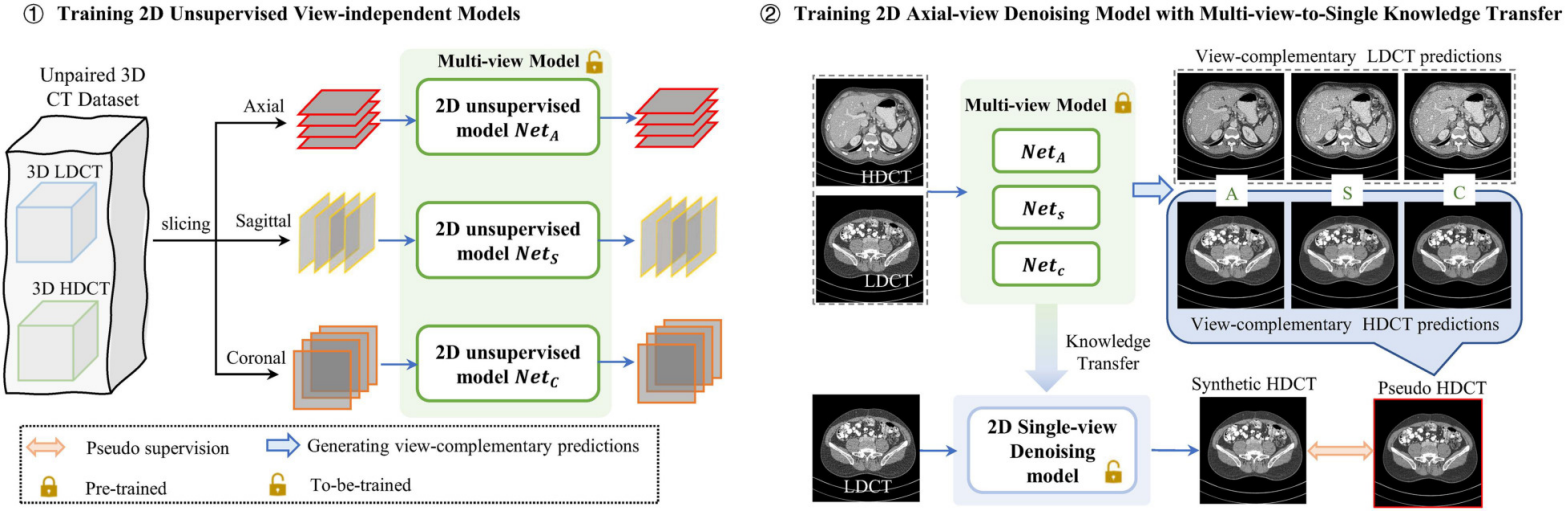
Proposed MvSKT framework for unpaired LDCT denoising consisting of two stages. In the first stage, a multi-view model comprising three CycleGAN networks trained on different views independently generates multi-view predictions for each axial-view CT image. In the second stage, a single-view model is trained on the axial view using pseudo-supervision derived from aggregating these multi-view predictions via weighted fusion.

To address these issues, we introduce a Multi-view-to-Single Knowledge Transfer (MvSKT) method for unpaired LDCT denoising. Our proposed MvSKT framework consists of two stages: a multi-view model and a single-view model. The multi-view model comprises three unsupervised view-independent models, each learning bidirectional mappings between LDCT and HDCT using unpaired CT data from different views. By fusing the 2D results of each model into a volumetric format and re-splitting it into axial-view images, we obtain multiple view-complementary predictions for each axial CT image. This process allows the multi-view model to generate multiple view-complementary predictions for each axial CT image, as depicted in the upper right panel of Figure 1. We further propose a cycle-consistency-weighted method to fuse these predictions into reliable pseudo-CT images, thereby supervising the training of a single-view model. This approach efficiently and effectively transfers multi-view information from the large multi-view model to the single-view model. Our single-view model also employs CycleGAN to learn from both the multi-view model and real unpaired CT data.

The main contributions of this paper are as follows:

- We propose a novel MvSKT framework for unpaired LDCT denoising, which leverages complementary information from multiple views to enhance denoising performance.
- We introduce a new pseudo-CT image generation method to transfer multi-view knowledge to a single-view model, utilizing a cycle-consistency-weighted fusion method to aggregate multiple view-complementary predictions into reliable pseudo-CT images.
- Extensive experimental results suggest the superiority of our method over other unpaired denoising methods and highlight its potential for practical clinical applications.

## 2 Related work

### 2.1 Paired low-dose CT denoising

In most LDCT denoising studies, models are typically trained using the widely recognized AAPM-Mayo Clinic Low-Dose CT Grand Challenge dataset. These models learn a nonlinear mapping from LDCT to high-dose CT (HDCT) images in a supervised fashion (5, 14, 18, 23, 29, 30). For example, Chen et al. introduced a residual encoder–decoder convolutional neural network (RED-CNN) (5). Yang et al. proposed leveraging Wasserstein GANs and perceptual similarity [18] to enhance visual quality. More recently, transformer-based networks have been employed for LDCT denoising, outperforming CNN-based methods (14, 23). Despite significant performance improvements achieved on the AAPM-Mayo dataset, these methods rely heavily on a large number of paired LDCT and HDCT images, which are often impractical to obtain in real-world settings.

### 2.2 Unpaired low-dose CT denoising

Unsupervised/self-supervised learning methods can eliminate the dependence on paired data (9, 13, 19, 20, 22, 26, 31–33). Among these, CycleGAN-based unsupervised methods have been particularly effective for LDCT denoising, including CycleGAN (13), Cycle-Circle (20, 28), and IdentityGAN (9). These methods are enhanced with various loss functions to improve performance. In addition, several self-supervised methods developed in the broader image processing field, such as Noise2Noise (27) and Noise2Void (26), have also been applied to CT denoising. These unpaired LDCT denoising methods primarily utilize single-view (axial) information for 2D denoising. However, single-view information may be insufficient for effectively distinguishing complex noise patterns from critical structures and tissues that must be preserved, especially in scenarios lacking strong supervision from paired data. In contrast, multi-view models could outperform single-view models in 2D LDCT denoising by providing richer and more diverse view-level information. In this work, we aim to integrate multi-view learning with knowledge transfer to enhance the performance of unpaired single-view LDCT denoising. Our approach retains the flexibility of unpaired single-view models while achieving higher accuracy.

### 2.3 Multi-view learning

Multi-view learning is a well-established technique in medical imaging, with a rich history and broad recognition. It has been effectively applied to various tasks, including image segmentation (34, 35), image classification (34, 36), and object detection (37). For example, Zhou et al. introduced a semi-supervised multi-organ segmentation approach that utilizes multi-view co-training, where a teacher model is trained using multi-view information to generate pseudo-labels for unlabelled data (38). Similarly, Xia et al. proposed an uncertainty-aware multi-view co-training (UMCT) framework for volumetric medical image segmentation (35). Inspired by these significant advancements, we aim to harness multi-view learning to gain deeper insights into the complex noise characteristics present in LDCT images and enhance noise reduction. Most works often obtain final results through multi-view fusion (37) or within a unified framework that employs multi-view co-training (35, 38). These methods, while effective, tend to increase inference time or complicate the training process. In contrast, our approach presents a two-stage framework that initially trains a multi-view model followed by a single-view model. A pseudo-supervision technique is employed to transfer multi-view knowledge from the multi-view model to the single-view model for unpaired LDCT denoising. This strategy not only boosts performance but also maintains efficiency without increasing inference time.

## 3 Methods

### 3.1 Overview

Our objective is to transform an arbitrary axial-view LDCT image x into its corresponding HDCT image y with significantly reduced noise. Recognizing that noise characteristics in 3D CT data vary across different views, we propose a MvSKT framework. As depicted in Figure 1, our framework operates in a strictly two-stage manner. In Stage 1 (pre-training), our multi-view model incorporates three 2D CycleGAN networks (13), which are fully pre-trained independently in an unsupervised manner using unpaired CT images from various views. Once the models converge, by generating multiple view-complementary predictions, the multi-view model serves as a stable foundation for Stage 2 (knowledge transfer), where a single-view model is trained to perform LDCT denoising specifically in the axial view.

### 3.2 Training a multi-view model with unpaired data

By splitting unpaired 3D anisotropic LDCT and HDCT data into saggital (S), coronal (C), and axial (A) views, we obtain three sets of unpaired 2D slices, denoted $\{X_{n}^{V};Y_{n}^{V}{\}}_{n=1}^{N_{V}}$, where $N_{V}$ represents the number of 2D slices obtained from the view V (V∈{A,S,C}). $X_{n}^{V}$ and $Y_{n}^{V}$ correspond to the LDCT and HDCT images from view V, respectively. As shown in Figure 1, three models, i.e., ${Net}_{V}$ (V∈{A,S,C}), are trained using unpaired CT images from the axial, sagittal, and coronal views, respectively. Each model is built on the 2D CycleGAN framework, which includes two mappings, i.e., LDCT-to-HDCT and HDCT-to-LDCT. In the following sections, we first provide a brief review of CycleGAN, then introduce the training process for a single model, and finally summarize the multi-view prediction generation process.

#### 3.2.1 Review of CycleGAN

A typical CycleGAN model, as described by Li et al. (13), consists of two generators, $G_{X\Rightarrow Y}$ and $G_{Y\Rightarrow X}$, and two discriminators, $D_{X}$ and $D_{Y}$. Considering an image translation model $Net_{V}$, the generator $G_{X\Rightarrow Y}$ is designed to translate an LDCT image into its corresponding HDCT image with reduced noise, while the generator $G_{Y\Rightarrow X}$ performs the reverse translation from HDCT to LDCT. $D_{X}$ distinguishes between real LDCT images and those generated by $G_{Y\Rightarrow X}$; similarly, $D_{Y}$ differentiates between real HDCT images and those generated by $G_{X\Rightarrow Y}$. To integrate these networks into a bidirectional mapping framework, a cycle-consistent loss is introduced. Specifically, the forward cycle involves passing the LDCT image through $G_{X\Rightarrow Y}$ to synthesize an HDCT image, which is then processed by $G_{Y\Rightarrow X}$ to reconstruct the LDCT image. The objective is for the reconstructed image to match the original input LDCT image. The backward cycle is defined analogously.

#### 3.2.2 Learning from unpaired data

To enable the network to learn from unpaired LDCT and HDCT data, we employ both cycle-consistent loss and GAN loss functions.

##### 3.2.2.1 Cycle-consistent loss

For unpaired LDCT and HDCT images {X,Y}, cycle-consistent losses are used to ensure structural consistency between the input and output of the generators. Specifically, forward cycle consistency requires that $x\to G_{X\Rightarrow Y}(x)\to G_{Y\Rightarrow X}(G_{X\Rightarrow Y}(x))\approx x$, while backward cycle consistency requires that $y\to G_{Y\Rightarrow X}(x)\to G_{X\Rightarrow Y}(G_{Y\Rightarrow X}(y))\approx y$. The cycle-consistent loss function is thus formulated as follows in Equation 1:

$$L_{CYC}=E_{x}{\left\|G_{Y\Rightarrow X}(G_{X\Rightarrow Y}(x))-x\right\|}_{1}+\;E_{y}{\left\|G_{X\Rightarrow Y}\;(G_{Y\Rightarrow X}(y))-y\right\|}_{1}s$$ (1)

##### 3.2.2.2 GAN loss

In addition, we incorporate adversarial learning to constrain the training procedure, encouraging the generators to produce samples that are indistinguishable from real data as assessed by the discriminator (39). For the generator $G_{X\Rightarrow Y}$ and its discriminator $D_{Y}$, we adopt LSGAN (40) in our work to stabilize the adversarial training process, which can be expressed as follows in Equation 2:

$$\begin{array}{cc} L_{GAN}(D_{Y}) & =\frac{1}{2}\left[E_{y}(D_{Y}(y)-1{)}^{2}+E_{x}(G_{X\Rightarrow Y}(x){)}^{2}\right]\\ L_{GAN}(G_{X\Rightarrow Y}) & =\frac{1}{2}\left[E_{x}(G_{X\Rightarrow Y}(x)-1{)}^{2}\right]\; \end{array}$$ (2)

Similarly, for the generator $G_{Y\Rightarrow X}$ and its discriminator $D_{X}$, the objective is defined in Equation 3:

$$\begin{array}{cc} L_{GAN}(D_{X}) & =\frac{1}{2}\left[E_{y}(D_{X}(x)-1{)}^{2}+E_{y}(G_{Y\Rightarrow X}(y){)}^{2}\right]\\ L_{GAN}(G_{Y\Rightarrow X}) & =\frac{1}{2}\left[E_{y}(G_{Y\Rightarrow X}(y)-1{)}^{2}\right]\; \end{array}$$ (3)

Overall, our final objective function is formulated as follows in Equation 4:

$$\begin{array}{c} D_{X}^{*},D_{Y}^{*}=\arg\min_{D_{X}}L_{GAN}(D_{X})+\arg\min_{D_{Y}}L_{GAN}(D_{Y})\\ \;G_{X\Rightarrow Y}^{*},G_{Y\Rightarrow X}^{*}=\arg\min_{G_{X\Rightarrow Y}}L_{GAN}(G_{X\Rightarrow Y})\\ \;+\arg\min_{G_{Y\Rightarrow X}}L_{GAN}(G_{Y\Rightarrow X})+\lambda \arg\min_{G_{X\Rightarrow Y},G_{Y\Rightarrow X}}L_{CYC} \end{array}$$ (4)

where λ is used to balance the importance between the losses. In our work, we experimentally set λ to 10.

#### 3.2.3 View-complementary prediction generation

**Figure 2:**
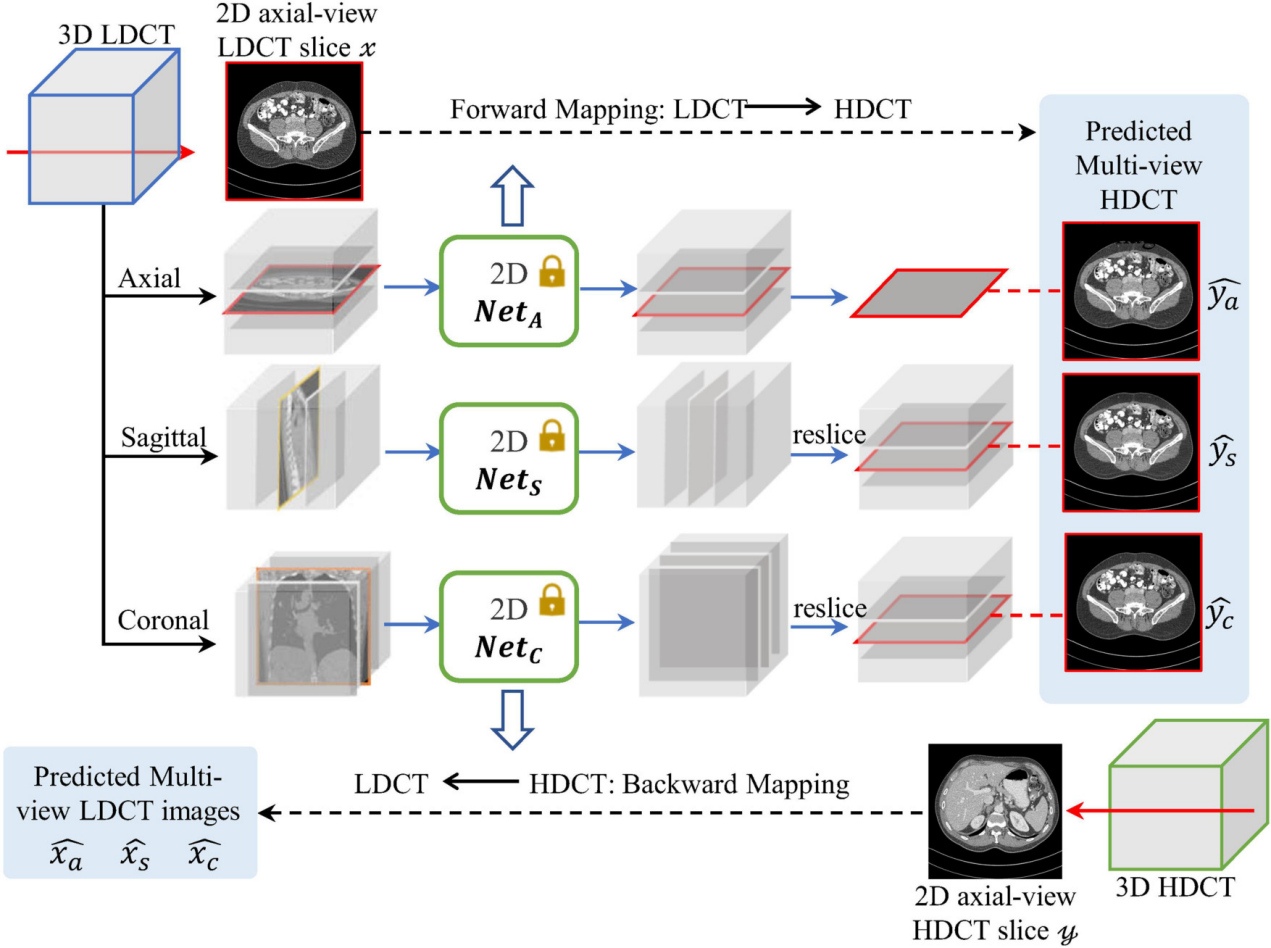
Example of generating multiple HDCT images containing view-specific information ($\hat{y^{a}}$,$\hat{y^{s}}$, $\hat{y^{c}}$) from the pre-trained axial-, sagittal-, and coronal-view denoising sub-models (i.e., ${Net}_{A}$, ${Net}_{S}$, ${Net}_{C}$) from our multi-view model.

Given a 3D LDCT volume, all models ${Net}_{A}$, ${Net}_{S}$, and ${Net}_{C}$ can generate its corresponding 3D HDCT volumes by stacking successive 2D results from the axial, sagittal, and coronal views, respectively. As depicted in Figure 2, we can obtain the corresponding multiple view-complementary HDCT predictions (i.e., $\hat{y_{a}},\hat{y_{s}},\hat{y_{c}}$) for an axial-view LDCT image x. Similarly, for an axial-view HDCT image y, we can derive the corresponding view-complementary LDCT predictions (i.e., $\hat{x_{a}},\hat{x_{s}},\hat{x_{c}}$) by stacking the successive 2D results generated by the different view-specific denoising sub-models of our multi-view model into a volumetric format and re-splitting the 3D results into an axial view. Note that x and y are unpaired. Consequently, the three models trained on different views can generate three predicted HDCT/LDCT images for each LDCT/HDCT axial image, as shown in the top-right panel of Figure 1. Since these predicted HDCT/LDCT images are generated by models trained on different views, we think that they contain view-specific information and provide complementary denoising information. For clarity, we denote them as view-complementary predictions.

Given the bidirectional translation capability of $Net_{V}$, we can associate real LDCT axial images with the view-complementary predictions for HDCT (denoted $\left\{X;\hat{Y_{a}},\hat{Y_{s}},\hat{Y_{c}}\right\}$), and real HDCT axial images with the view-complementary predictions for LDCT (denoted $\left\{Y;\hat{X_{a}},\hat{X_{s}},\hat{X_{c}}\right\}$). Leveraging these pre-generated view-complementary predictions, a single-view model is trained exclusively on the axial view using pseudo-supervision. This involves a cycle-consistency-weighted fusion strategy for the multiple view-complementary predictions to generate reliable pseudo-CT images. Note that we train the single-view model on the axial view rather than the sagittal or coronal views, in accordance with clinical CT imaging practices and reading conventions.

### 3.3 Training a single-view model with Multi-view-to-Single Knowledge Transfer

In this study, we propose to transfer multi-view knowledge from a multi-view model to a single-view model. Initially, we generate two sets from unpaired LDCT and HDCT data, including paired real LDCT axial images and view-complementary predictions for HDCT $\left\{X;\hat{Y_{a}},\hat{Y_{s}},\hat{Y_{c}}\right\}$ and LDCT $\left\{Y;\hat{X_{a}},\hat{X_{s}},\hat{X_{c}}\right\}$. Intuitively, predictions fused from multiple views are expected to be superior to those derived from a single view. Therefore, our goal is to produce reliable pseudo-LDCT and -HDCT images from multiple view-complementary predictions to supervise the training of the single-view model. We introduce a cycle-consistency-weighted fusion method to aggregate the multiple view-complementary predictions into trustworthy pseudo-images. This approach enhances the training of our single-view model for axial-view LDCT image denoising. The following sections discuss our proposed cycle-consistency-weighted fusion method and the training procedure for the single-view model.

#### 3.3.1 Cycle-consistency-weighted fusion

To fuse multiple view-complementary predictions, a straightforward approach is to apply average fusion. However, due to inter-view differences, average fusion may not yield an optimal combination of various views. In our work, we propose a cycle-consistency-weighted fusion method that leverages the cycle-consistent generation within the CycleGAN framework (13). Specifically, we continue to employ a CycleGAN as our single-view model. Based on cycle-consistent generation, we reasonably assume that the closer a reconstructed image is to the estimation of the input image, the more reliable the predicted image is. For the view-complementary predictions of HDCT (i.e., $\hat{y_{a}}$, $\hat{y_{s}}$, and $\hat{y_{c}}$) corresponding to a real LDCT image x, we can feed them into the $G_{Y\Rightarrow X}$ to obtain the respective reconstructed LDCT images. By comparing the reconstructed LDCT images with the real LDCT image, we compute the view weight map of each predicted HDCT. This process can be formulated as follows:

$$\begin{array}{rl} {\text{diff}}_{a} & ={\left\|G_{Y\Rightarrow X}(\hat{y^{a}})-x\right\|}_{1},\;{\text{diff}}_{s}={\left\|G_{Y\Rightarrow X}(\hat{y^{s}})-x\right\|}_{1},\;\\ {\text{diff}}_{c} & ={\left\|G_{Y\Rightarrow X}(\hat{y^{c}})-x\right\|}_{1},\\ \text{Diff} & ={\text{diff}}_{a}+{\text{diff}}_{s}+{\text{diff}}_{c} \end{array}$$ (5)

as well as

$$\begin{array}{rl} w_{s}^{h} & =\frac{1}{2}\;*\;({\text{diff}}_{a}+{\text{diff}}_{c})/\text{Diff},\;w_{c}^{h}=\frac{1}{2}\;*\;({\text{diff}}_{a}+{\text{diff}}_{s})/\text{Diff},\\ w_{a}^{h} & =1-w_{s}^{h}-w_{c}^{h} \end{array}$$ (6)

Equation 5 calculates the difference map between the LDCT image reconstructed from the view-specific HDCT estimation and the real LDCT image x. The view weight maps for each prediction, i.e., $\left\{w_{a}^{h},w_{s}^{h},w_{c}^{h}\right\}$, are computed by Equation 6. Similarly, given the view-complementary predictions for LDCT $\left\{\hat{x_{a}},\hat{x_{s}},\hat{x_{c}}\right\}$, we also calculate the respective view weight maps, i.e., $\left\{w_{a}^{l},w_{s}^{l},w_{c}^{l}\right\}$, in the same manner as the calculation of $\left\{w_{a}^{h},w_{s}^{h},w_{c}^{h}\right\}$. With the view weight maps, the cycle-consistency-weighted fusion can be expressed as follows in Equation 7:

$$\begin{array}{cc} \hat{y} & =w_{a}^{h}\;*\;\hat{y^{a}}+w_{s}^{h}\;*\;\hat{y^{s}}+w_{c}^{h}\;*\;\hat{y^{c}},\\ \hat{x} & =w_{a}^{l}\;*\;\hat{x^{a}}+w_{s}^{l}\;*\;\hat{x^{s}}+w_{c}^{l}\;*\;\hat{x^{c}}, \end{array}$$ (7)

where $\hat{y}$ and $\hat{x}$ denote the fused pseudo-HDCT and -LDCT images, respectively.

In general, our proposed cycle-consistency-weighted fusion method assigns relative importance to each view-specific prediction based on the cycle-consistent generation characteristic of our CycleGAN-based single-view model. Note that the view weight maps are computed dynamically at each step as the single-view model is trained.

#### 3.3.2 Axial-view LDCT image denoising

We train a single-view model to perform axial-view 2D LDCT image denoising by leveraging multiple sets, including the unpaired CT data {X;Y}, paired real LDCT and pseudo-HDCT images $\left\{X;\hat{Y}\right\}$, and paired real HDCT and pseudo-LDCT images $\left\{Y;\hat{X}\right\}$. The pseudo-images are generated from multiple view-complementary predictions using our proposed cycle-consistency-weighted fusion strategy during each training iteration of the single-view model. Therefore, for training our single-view model, in addition to the cycle-consistent loss and GAN loss used in the standard CycleGAN framework, a pseudo-supervised loss is further used to constrain the output of each generator to match the corresponding pseudo-image. The pseudo-supervised loss for $G_{X\Rightarrow Y}$ and $G_{Y\Rightarrow X}$ can be expressed as follows in Equation 8:

$$\begin{array}{rl} L_{REC} & =E_{x}\;{\left\|(G_{X\Rightarrow Y}(x))-\hat{y}\right\|}_{1}\\ & \;+E_{y}\;{\left\|(G_{Y\Rightarrow X}(y))-\hat{x}\right\|}_{1} \end{array}$$ (8)

The total objective for two generators in our single-view model can be formulated as follows in Equation 9:

$$L_{G}=\lambda_{1}L_{GAN}+\lambda_{2}L_{CYC}+\lambda_{3}L_{REC}$$ (9)

We experimentally set the balance factors $\lambda_{1}$, $\lambda_{2}$, and $\lambda_{3}$ as 1, 10, and 5, respectively. In addition, the LSGAN (40) is also used to optimize the two discriminators in our single-view model.

### 3.4 Implementations

#### 3.4.1 Network architecture

In our multi-view model, each sub-network employs the same network architecture. The generator of each CycleGAN-based sub-network comprises a down-sampling encoder module, a transfer residual module, and an up-sampling decoder module. We refer to the generator as ResCNN. In our work, the network architecture of the generator in the multi-view model is not carefully selected or customized. Alternative architectures, such as U-Net (41) or ResUnet (42), can also be used. The encoder module consists of three Conv-IN-ReLU blocks, each with a convolution layer followed by “Instance Normalization” and “ReLU” activation. The number of channels in these blocks is 32, 64, and 128, respectively. The stride of the last two blocks is set to 2 to downsample the feature maps and encode information from the latent space with a larger receptive field. The transfer residual module consists of six residual blocks. In the decoder, two Deconv-IN-ReLU blocks are used to restore the resolution to the original one, followed by a convolution layer to output the network prediction. The kernel size is 3×3 for all convolution and deconvolution layers, except the first and last convolution layers, which use a kernel size of 7×7.

For our single-view model, we adopt the same network architecture as RED-CNN (5) for the generators. RED-CNN utilizes fully convolutional layers without downsampling or upsampling, thus reducing resolution loss, although it has a small receptive field. With pseudo-supervision for our single-view model, we found that a network with minimal resolution loss can better reconstruct the fine structures. For both the multi-view and single-view models, all discriminators are implemented using the PatchGAN discriminator (43).

#### 3.4.2 Training details

In this paper, we utilize four CycleGAN-based networks. Our multi-view model comprises three CycleGAN sub-networks, each trained on different views in an unsupervised manner. In addition, our single-view denoising model is trained on the axial view under pseudo-supervision provided by our multi-view model. During training, the two generators and two discriminators are trained in an alternative manner; i.e., the generators are trained while keeping the discriminators fixed, followed by training the discriminators while keeping the generators fixed. We employ the Adam optimizer with a learning rate of $1\times 10^{-5}$ to optimize all networks. A patch-based training strategy is applied across all networks. The input LDCT/HDCT images are cropped to a size of 256×256 for the ResCNN generators used in the multi-view model and to 64×64 for the RED-CNN (5) generators used in the single-view model. The batch sizes for the multi-view and single-view models are 5 and 3, with 2 and 10 patches sampled per iteration, respectively. All models are trained for 100 epochs. We implement our method using PyTorch. All experiments are conducted on a workstation equipped with a V100 GPU. Notably, our method does not introduce additional computation overhead after training, as only the single-view model is used in the inference phase. Our code, including data preprocessing, model training, and evaluation, will be made publicly available at https://github.com/Candyeeee/MvSKT.

## 4 Experiments

### 4.1 Experimental data

#### 4.1.1 AAPM-Mayo dataset

This dataset comprises paired abdominal HDCT (full-dose) and LDCT (quarter-dose) data from 10 anonymous subjects. The LDCT data are simulated by inserting Poisson noise into the projection data. For training our unsupervised models on both multiple-view and single-view (i.e., axial view), we split the original 10 cases into eight cases for training and two cases for testing. When training our unsupervised models on multiple views, we first split the 3D data of the training and test sets into axial, coronal, and sagittal views. We then shuffle the training data to remove pairing between the LDCT and HDCT images, while the test data remain paired for quantitative evaluation. This results in a total of 5,092/844, 4,096/1,024, and 4,096/1,024 view-specific CT images available for training/testing the unsupervised models on the axial, sagittal and coronal views, respectively. Our single-view model is trained on unpaired axial-view data, with the training and test sets comprising 5,092 and 844 images, respectively.

### 4.2 Ablation study

#### 4.2.1 Effect of multi-view learning and knowledge transfer

To assess the effectiveness of multi-view learning and knowledge transfer in our method, we compare the quantitative performance of multi-view models with various view-specific model combinations against our single-view model on the AAPM-Mayo dataset. We regard the model trained on a single axial view in an unsupervised manner using CycleGAN (13) as the baseline method. In addition to the baseline method, we compare four multi-view models with different view-specific model combinations to our single-view model trained with pseudo-supervision. For clarity, detailed descriptions of all methods are provided in Table 1. In Table 1, “Our-M” with “A+S” views denote our multi-view model consisting of two sub-networks trained on axial and sagittal views, respectively, and the results correspond to the average performance of these two models. Other variant models are similarly defined. “Our-S” refers to our single-view model trained with pseudo-supervision. All models follow the same data splitting and training strategy described earlier. We evaluate this group of experiments under two settings. In setting 1, the generator of each model is built on “ResCNN” in the baseline and Our-M variants, while on “RED-CNN” in the Our-S variant. In setting 2, the generators of all models are built on “RED-CNN.” Three commonly used metrics are employed to quantitatively assess the image quality of denoised images: (1) relative mean absolute error (RMAE), (2) peak signal-to-noise ratio (PSNR), and (3) structure similarity (SSIM) index.

**Table 1:** Quantitative analysis of the multi-view learning and knowledge transfer used in our proposed method.

Method	View	KT	SSIM	PSNR	RMAE	SSIM	PSNR	RMAE
Baseline	A	No	0.9275 ± 0.040	40.15 ± 1.854	30.43 ± 8.592	0.9526 ± 0.038	42.19 ± 2.057	24.04 ± 8.592
Our-M	A+S	No	0.9394 ± 0.029	40.87 ± 1.711	27.89 ± 5.594	0.9556 ± 0.019	42.58 ± 1.862	23.00 ± 5.015
Our-M	A+C	No	0.9297 ± 0.027	40.93 ± 1.670	27.72 ± 5.535	0.9553 ± 0.019	42.49 ± 1.872	23.25 ± 5.086
Our-M	S+C	No	0.9339 ± 0.020	40.84 ± 1.418	27.82 ± 4.641	**0.9565 ± 0.018**	42.62 ± 1.833	22.87 ± 4.892
Our-M	A+S+C	No	0.9388 ± 0.024	41.33 ± 1.653	26.44 ± 5.160	0.9564 ± 0.018	42.64 ± 1.876	22.84 ± 5.001
Our-S	A+S+C	Yes	**0.9565 ± 0.018**	**42.80 ± 1.796**	**22.40 ± 4.723**	0.9554 ± 0.018	**42.73 ± 1.819**	**22.58 ± 4.814**

“A,” “S,” and “C” denote the axial, sagittal, and coronal views, respectively. “KT” denotes “knowledge transfer.” “Our-M” and “Our-S” denote our multi-view and single-view models, respectively. The generators of the baseline and Our-M variants are built on “ResCNN” in the dark part, while on “RED-CNN” in the blue part. The generators of our-S models in both the dark and blue parts adopt the “RED-CNN” network.

Bold values indicate the best results.

The quantitative results are summarized in Table 1. From Table 1, we have the following observations. First, the “Our-M” models with arbitrary view combinations outperform the baseline method, which utilizes only the single axial view. Second, the “Our-S” model, which transfers multi-view knowledge from multi-view model, achieves performance that is better than or comparable to the multi-view models. For instance, when using “ResCNN” as the generator, it enhances the SSIM and PSNR by 1.77 percentage points and 1.47 dB and reduces the RMAE by 4.04 compared to “Our-M” with “A+S+C” views. Using “RED-CNN” as the generator, although the SSIM metric of “Our-S” is slightly lower than that of the “Our-M” variants, “Our-S” achieves the highest PSNR and the lowest RMAE. In addition, we observe that “Our-M” and its variants perform better when the generators in their sub-models are built on “RED-CNN” rather than on “ResCNN.” However, when “Our-S” uses the multi-view knowledge transferred from “Our-M,” “Our-S” performs slightly better when the generator of each sub-model of “Our-M” is built on “ResCNN” rather than on “RED-CNN.” A possible explanation is as follows: the respective field for “RED-CNN” is limited because it consists of fully convolutional layers without any downsampling or upsampling. Consequently, “Our-M” built on “RED-CNN” cannot capture view difference information well to provide “Our-S.” In contrast, “ResCNN” with its larger respective field can better capture diverse information for each view-specific sub-model in the multi-view model. Thus, in the proposed MvSKT, we adopt the “ResCNN” generator for our multi-view model. To allow for pseudo-supervision, we adopt the RED-CNN generator with reduced resolution loss for our single-view model to facilitate the reconstruction of fine structures. We chose RED-CNN and ResCNN due to their effectiveness with our dataset. While advanced models like transformers have shown promising results, our experiments favoured RED-CNN and ResCNN for our dataset size. We speculate that transformers might perform better with large-scale data, indicating a path for future research. In this work, our focus is to showcase the effectiveness of multi-view learning and knowledge transfer for LDCT denoising, which we believe significantly advances CT image denoising and could enhance diagnostic quality and reduce patient radiation exposure.

**Figure 3:**
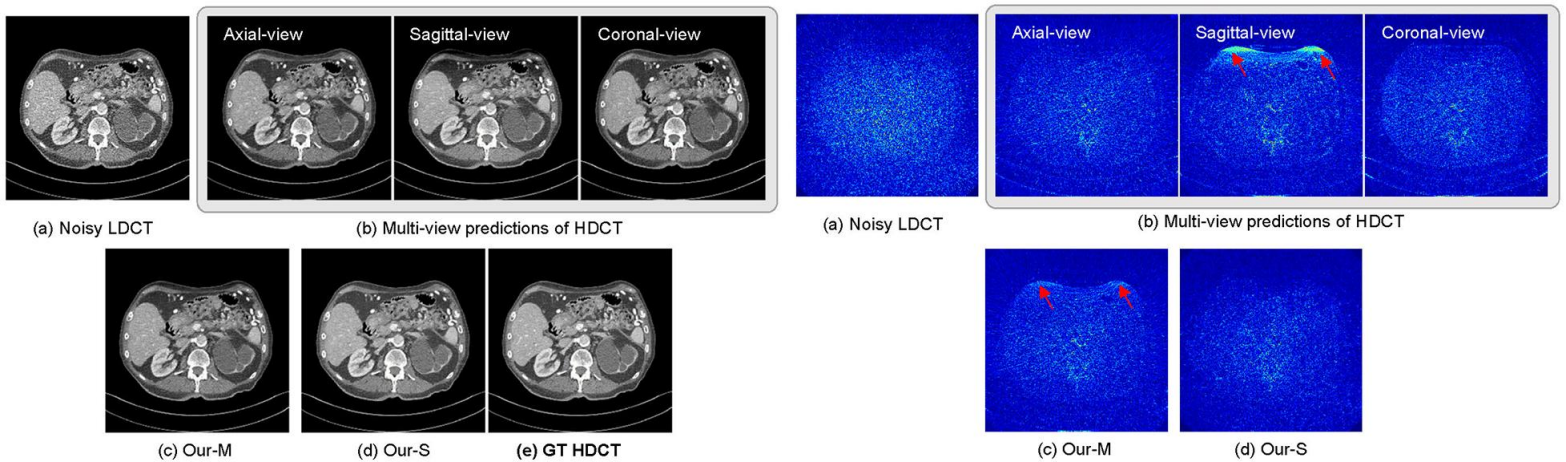
Visual example of view-complementary predictions of HDCT and the HDCT estimations generated by Our-M and Our-S models. The view-complementary predictions include three HDCT images generated from unsupervised models trained on axial, sagittal, and coronal views, respectively. Our-M represents the average fusion of these predictions, while Our-S shows the result from our single-view model. The right panel displays the difference maps between the ground-truth (GT) HDCT and other images.

Furthermore, we observe that the reconstruction quality varies across individual views depending on the specific slice and patient anatomy. When a particular view yields a suboptimal prediction in a localized region—such as the edge artefacts observed in the sagittal view for the specific slice shown in Figure 3 (indicated by red arrows)—it inevitably degrades the performance of a naively averaged ensemble (Our-M). However, our proposed knowledge transfer framework effectively mitigates this issue by utilizing the cycle-consistency error as a dynamic, view-agnostic measure of anatomical reliability. Regardless of which view underperforms, if a pre-trained generator struggles with a complex anatomical region and produces artefacts, it naturally incurs a higher local cycle-consistency error. Our dynamic fusion mechanism detects this signal and automatically down-weights the specific unreliable view for those pixels, intelligently shifting the weights towards the other, more reliable views. Consequently, our final single-view student model (Our-S) avoids these localized artefacts seen in the average ensemble and consistently preserves sharp, accurate boundaries.

These results demonstrate the effectiveness of the multi-view learning and knowledge transfer employed in our proposed MvSKT method for 2D LDCT image denoising. Note that training a single-view model took approximately 23 h on a computer machine equipped with a 3,090 GPU, and testing a single slice at a resolution of 512*512 took 0.08 s. While training three teachers increases upfront cost, we emphasize that this is a one-time investment. The student model’s inference speed (0.08 s/slice) is three times faster than multi-view baselines (0.25 s/slice for axial + sagittal + coronal fusion), thus avoiding any additional computational overhead in practical applications.

#### 4.2.2 Effect of cycle-consistency-weighted fusion

To assess the efficacy of our proposed cycle-consistency-weighted fusion strategy, we compare it with a straightforward and common approach that averages multi-view predictions to produce reliable pseudo-CT images for training our single-view model. We generate view-complementary predictions by training two multi-view models, such as “Multi-view Model 1” and “Multi-view Model 2.” The generator of each sub-model is built on “ResCNN” in “Multi-view Model 1,” while on “RED-CNN” in “Multi-view Model 2.” Then, we train our single-view models, such as “Our-S Model 1” and “Our-S Model 2,” using pseudo-supervision from “Multi-view Model 1” and “Multi-view Model 2” via two fusion methods, respectively.

**Figure 4:**
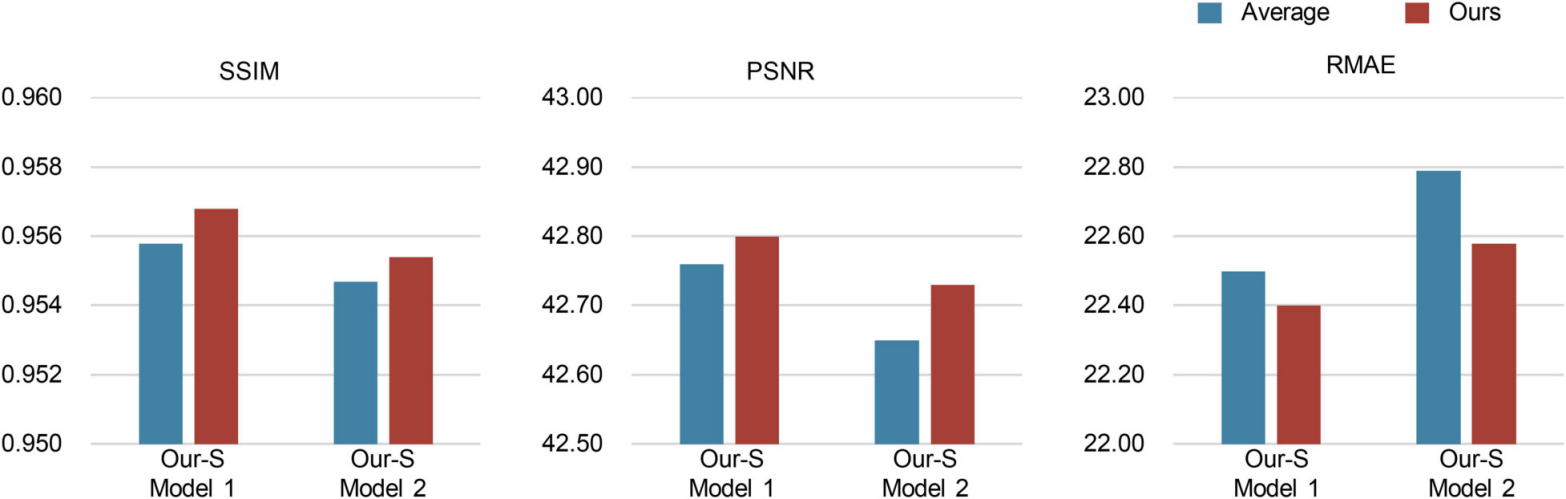
Performance comparison of the single-view models using average fusion and our proposed cycle-consistency-weighted fusion.

The quantitative results on the AAPM-Mayo test set are depicted in Figure 4. From Figure 4, it is evident that the proposed fusion strategy is slightly better than the straightforward average fusion for LDCT denoising in both settings. These results indicate that our proposed cycle-consistency-weighted method can help the single-view model learn more reliable information from multi-view predictions than average fusion.

### 4.3 Comparison with other methods

In this section, we compare our proposed method with several existing methods for LDCT denoising on the AAPM-Mayo dataset, including the typical non-learning-based BM3D method (44), the unsupervised deep learning-based methods such as the baseline CycleGAN (13) and IdentityGAN (9), and the self-supervised Noise2Void (26) and Noise2Sim (45) methods. In addition, the to-be-tested LDCT and a classic supervised deep-learning method, REDCNN (5), are used as the lower and upper bounds, respectively. Therein, IdentityGAN introduced an identity loss based on the CycleGAN framework. We implemented IdentityGAN following the original paper, keeping the weighted combination of all training loss terms consistent with Kang et al. (9). To be fair, CycleGAN and IdentityGAN use the same network architecture, “RED-CNN,” for their generators as in our single-view model. Noise2Void is a self-supervised training method that requires only a single body of noisy images for denoising models. Noise2Sim is a similarity-based self-supervised deep denoising approach that operates in a non-local and non-linear manner to suppress independent and correlated noise. We implemented the Noise2Void and Noise2Sim methods according to the codes released by their authors, which are available at https://github.com/juglab/n2v and https://github.com/niuchuangnn/noise2sim, respectively. Note that all methods have the same data splitting for training and testing as in our MvSKT.

**Figure 5:**
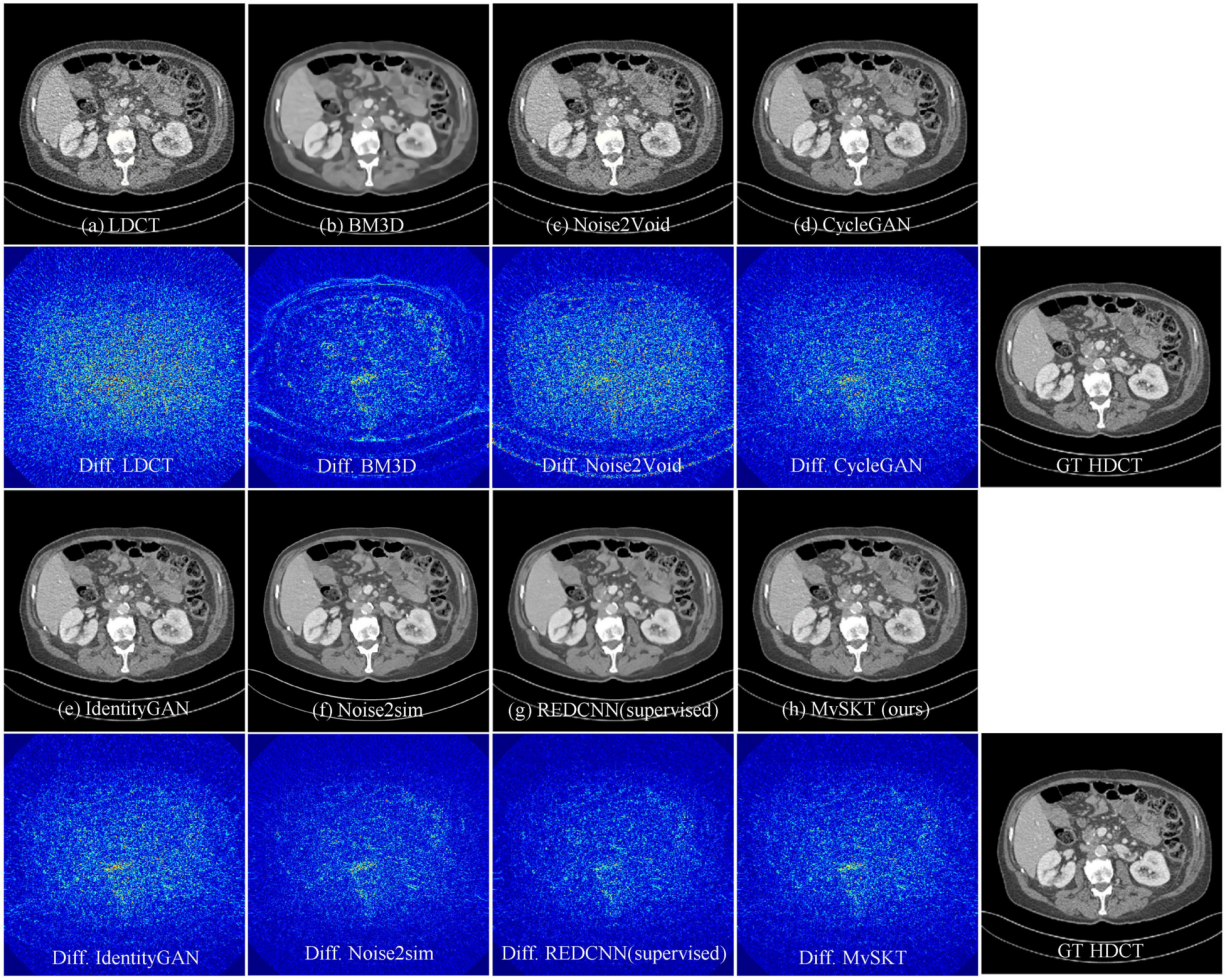
Qualitative comparison of different methods on case L109 from the AAPM-Mayo dataset. The difference maps between each method and the ground-truth HDCT image are shown for a better view. The displayed window is [−240, 300] HU.

The visual denoised results for all methods are presented in Figure 5, and the quantitative comparisons on the AAPM-Mayo test set are detailed in Table 2. From Figure 5, we observe that the BM3D (44) results are over-smoothed, losing significant fine-textural and structural details, while N2V reduces noise minimally. In contrast, methods such as CycleGAN, IdentityGAN, REDCNN, and our proposed MvSKT achieve a better balance between noise reduction and structure preservation by leveraging additional HDCT data. Despite the denoised images from CycleGAN, IdentityGAN, and our method appearing similar due to their shared network architecture, our method demonstrates superior noise reduction performance by leveraging multi-view learning and knowledge transfer. Compared with other methods that do not require paired data, MvSKT closely approaches the supervised RED-CNN method across all metrics. Notably, Noise2Sim also yields appealing results, with SSIM values close to those achieved by the supervised RED-CNN method. However, it performs worse on PSNR and RMAE metrics than our proposed method due to its quasi-supervised nature, which may lead to over-smoothing and the loss of some signals. We calculated the p-values for the metric differences between our method and other competing methods. All resulting p-values are less than 0.05, indicating that the observed differences are statistically significant at the 95% confidence level.

**Table 2:** Quantitative comparison (mean ± SD) of different methods on the AAPM-Mayo test set.

Method	SSIM	PSNR	RMAE
LDCT	0.9049 ± 0.038	38.82 ± 2.057	35.61 ± 8.592
BM3D (44)	0.9445 ± 0.011	40.65 ± 0.826	28.18 ± 2.639
CycleGAN (13)	0.9526 ± 0.038	42.19 ± 2.057	24.04 ± 8.592
IdentityGAN (9)	0.9531 ± 0.019	42.44 ± 1.774	23.31 ± 4.853
Noise2Void (26)	0.9190 ± 0.028	38.63 ± 1.346	35.83 ± 5.688
Noise2Sim (45)	0.9622 ± 0.014	42.00 ± 3.104	26.02 ± 13.931
REDCNN (5)	**0.9642 ± 0.013**	**43.72 ± 1.570**	**20.00 ± 3.667**
MvSKT	0.9565 ± 0.018	42.80 ± 1.796	22.40 ± 4.723

Bold values indicate the best results.

## 5 Discussion

This paper introduces a novel method, the MvSKT framework, for 2D low-dose CT denoising without paired data.

### 5.1 Strengths of the proposed MvSKT

In both quantitative and qualitative comparisons, our proposed MvSKT framework demonstrates reduced over-smoothing issue compared to the classic BM3D method (44), and even the supervised method REDCNN (5). In addition, our method outperforms CycleGAN (13), IdentityGAN (9), and Noise2Void (26) in terms of denoising effectiveness. The primary reason for performance improvement in our work is the introduction of an effective and efficient knowledge transfer framework that transfers multi-view knowledge to the single-view model. Multi-model ensembles can achieve better results than a single model; however, if one model performs poorly, it can negatively impact the ensemble results, as illustrated in Figure 3. Furthermore, inferring multiple models imposes an additional computational burden. In contrast, we propose leveraging the results of multiple models in the multi-view model to retrain a single-view model, enabling the single-view model to automatically learn valid information from the multi-view model and discard incorrect information. Moreover, our single-view model is more time- and computationally efficient than deploying multiple models.

### 5.2 Comparison with 2.5D and 3D methods

To evaluate the tradeoffs of spatial context modelling, we compared our approach with a hybrid 2.5D method (2.5D REDCNN) and 3D models (46), as reported in Table 3. Surprisingly, the supervised 2.5D REDCNN did not outperform the standard 2D REDCNN, indicating that merely stacking adjacent slices offers limited empirical advantage for this specific task. Furthermore, extending 2.5D or 3D architectures to unsupervised learning introduces severe training instability. Mapping complex joint distributions across consecutive slices without paired ground truth is highly ill-posed, which also explains why the 3D unsupervised CycleGAN underperforms our MvSKT model. In addition, both 2.5D and 3D models inevitably increase deployment complexity by requiring multi-slice buffering during inference. In contrast, our MvSKT effectively distills global multi-view 3D correlations into a strictly single-slice 2D inference architecture, offering a superior tradeoff between denoising performance, training stability, and clinical deployment efficiency.

**Table 3:** Quantitative comparison with 2.5D and 3D methods on the AAPM-Mayo test set.

Method	Supervised	SSIM	PSNR	RMAE
REDCNN	Yes	0.9642 ± 0.013	43.72 ± 1.570	20.00 ± 3.667
3D ResCNN	Yes	0.9675 ± 0.013	43.84 ± 1.492	20.00 ± 2.622
2.5D REDCNN	Yes	0.9638 ± 0.015	42.98 ± 1.650	21.03 ± 4.527
3D CycleGAN	No	0.9511 ± 0.036	42.10 ± 2.058	24.21 ± 7.601
MvSKT	No	0.9565 ± 0.018	42.80 ± 1.796	22.40 ± 4.723

### 5.3 Limitation and future work

Our approach involves training multiple models; however, we do not elaborate on the network architecture of each model in this work. Specific network structures, such as transformer-based models, may better capture long-range dependencies between LDCT pixels for improved denoising. Optimizing network architectures for better performance is one of the directions to improve our current work and will be a focus of our future work. In addition, the clinical value of the denoised images generated by our model needs further validation. In the future, we plan to collect real, labelled clinical low-dose lung CT data to validate the clinical utility of our denoising model by applying it to downstream tasks such as disease diagnosis and lesion segmentation.

## 6 Conclusion

We propose a novel unpaired CT denoising framework that leverages multi-view learning and knowledge transfer. By introducing the MvSKT framework, our denoising model (i.e., our single-view model) exploits complementary information from multiple views of LDCT to generate high-quality images that balance noise reduction and structure preservation. This approach outperforms existing methods that rely solely on single axial-view information. We conducted quantitative and qualitative analyses of the public AAPM-Mayo dataset, demonstrating the superiority of our method over other competitive LDCT denoising techniques.

## Data Availability

Publicly available datasets were analyzed in this study. This data can be available at https://www.aapm.org/grandchallenge/lowdosect/.
